# Advanced Monitoring Systems Based on Battery-Less Asset Tracking Modules Energized through RF Wireless Power Transfer

**DOI:** 10.3390/s20113020

**Published:** 2020-05-26

**Authors:** Roberto La Rosa, Catherine Dehollain, Patrizia Livreri

**Affiliations:** 1 STMicroelectronics, Stradale Primosole 50, 95121 Catania, Italy; roberto.larosa@st.com; 2 Ecole Polytechnique Federale de Lausanne, 1015 Losanne, Switzerland; catherine.dehollain@epfl.ch; 3 Department of Engineering, University of Palermo, Viale delle Scienze Ed.9, 90128 Palermo, Italy

**Keywords:** wireless power transfer, wpt, wireless sensor networks, wsns, radio frequency, energy harvesting, wireless battery charger, internet of things

## Abstract

Asset tracking involving accurate location and transportation data is highly suited to wireless sensor networks (WSNs) featuring battery-less nodes that can be deployed in virtually any environment and require little or no maintenance. In response to the growing demand for advanced battery-less sensor tag solutions, this article presents a system for identifying and monitoring the speeds of assets in a WSN with battery-less tags that receive all their operating energy through radio frequency (RF) wireless power transfer (WPT) architecture, and a unique measurement approach to generate time-domain speed readouts. The assessment includes performance characteristics and key features of a system on chip (SoC) purposely designed to power a node through RF WPT. The result is an innovative solution for RF to DC conversion able to address the principal difficulties associated with maximum power conversion efficiency (PCE) with sensitivity and vice versa, a strategy, and a design optimization model to indicate the number of readers required for reliable asset identification and speed measurement. Model validation is performed through specific tests. Experimental results demonstrating the viability of the proposed advanced monitoring system are provided.

## 1. Introduction

The introduction of Internet of Things (IoT) technologies, connected and synchronized devices, and smart solutions has delivered unprecedented advancements in wireless sensor networks (WSNs), which represent an emerging set of technologies that promise to dramatically improve daily life [1]. Smart wireless sensor nodes are expected to pervade all the emerging applications in the Internet of Things (IoT) arena [2]. Indeed, many WSN platforms aimed at enhancing services, comfort, security, and energy saving have already been proposed for smart city and home and office automation scenarios [3,4,5,6,7,8,9]. WSNs also lend themselves very well to asset-monitoring applications though their ability to monitor and record the exact positions and transportation conditions of assets, personal objects, and the like [10]. Here, sensor nodes transmit small packets of information to indicate the presence, identity, position, and speed of a resource. The amount of data to be exchanged is low and the system does not require significant levels of power or bandwidth. An ideal tag for asset tracking is a non-disposable, inexpensive, and maintenance-free device that can be used almost anywhere [11,12,13]. An effective solution requires embedded communication and sensing capabilities, signal processing, power management, and power generation [14,15], which is far removed from the simple tag transponder functionality suitable only for short distance item identification. The wireless sensor node is now a more complex device capable of sensing, data analysis, and communication [16], which, however, implies a greater energy demand that will necessarily require a battery, with corresponding burdens on cost, maintenance, and miniaturization [17]. For this reason, the most telling performance characteristics of WSN nodes are power consumption and maximum throughput at maximum communication distance, in addition to other critical factors such as size and cost [2,5]. WSN nodes can implement energy-efficient communication schemes combined with low power designs to extend battery life to months or even years [2]. This is why wireless protocols that use unlicensed ISM bands (industrial, medical, and scientific frequency bands) such as ZigBee [18], Bluetooth and Bluetooth Low Energy (BLE) [19], are widely implemented in low-power WSN designs. The BLE protocol in particular offers reduced power consumption and easy setup and connection with smart devices [20,21,22]. Low energy consumption and energy efficiency are obtained through strategic hardware and firmware co-design, and comprehensive optimization based on the final application. Conventional battery-powered systems are not always the optimal solution because of the additional cost, weight, and size penalties that batteries introduce, not to mention longevity and maintenance issues. In addition, the potential use of battery and super capacitors poses problems regarding system power management [23,24]. Maintenance in WSNs is not just an expense concern; it can also involve complexities in terms of safety and access, and some environments may simply be too hot for reliable battery operation. In common working conditions [25], a substantial reduction in battery power consumption can be achieved by reducing or eliminating standby power [26,27,28,29,30,31,32,33,34], which can directly translate into longer system lifetime, further miniaturization, and reduced maintenance intervention frequencies. Maintenance of battery-powered nodes can also be facilitated by implementing over-the-distance wireless battery charging using radio frequency (RF) wireless power transfer (WPT) [35,36,37,38,39,40]. While these solutions can help alleviate maintenance and miniaturization problems, they cannot solve them altogether. Where feasible, such as in applications involving low duty-cycle sensors, it is far more preferable to implement battery-less devices, with the evident advantage of rendering them non-disposable, virtually unlimited in lifetime, more cost-efficient, and usable in environments where batteries can be dangerous [41,42,43,44,45]. For these reasons, battery-less solutions are gaining momentum [43,46,47,48,49], with engineers increasingly opting for regenerative energy harvesting (EH), including RF EH and WPT. Implementing efficient WPT and RF EH is not a simple task, because even though RF energy offers high pervasiveness and the ability to be directed to out-of-sight locations, its power conversion efficiency (PCE) remains very low. All this has already prompted many researchers to contribute highly enlightening papers on the subject [50,51,52,53,54,55,56,57,58,59,60,61,62,63,64,65,66,67]. This paper contributes to this field of research with an investigation of RF WPT between a power transmitter (reader) and an RF self-powered, battery-less BLE tag within a WSN infrastructure. It describes the challenges and provides solutions for the design of a typical asset tracking system in which assets are monitored using battery-less BLE tags. The tag travels at a certain velocity with respect to the reader in a dynamic context where the distance between the reader and the tag is not constant. The distinguishing elements in this research are that RF WPT is performed in a moving environment and BLE technology in employed for data communication. The focus of this investigation is to estimate the minimum number of readers required to continuously power a moving tag, and in doing so, demonstrate how a totally battery-less sensor that is self-powered through RF WPT can be part of a speed measurement system that produces time-domain readouts which can be transmitted via IoT mechanisms. The outcome is a strategy and a mathematical model for designing the infrastructure by providing the optimum number of readers needed to perform the identification of an asset and measure its speed. The key features, architecture, and performance characteristics of a system on chip (SoC) specifically designed to power a node by means of RF WPT are discussed in detail. Specific tests, simulations and experimental results are provided. The paper is organized as follows. Section 2 describes the architecture of the system in terms of the reader and the battery-less BLE asset tag. Section 3 deals with the design methodology for the WPT system with equations and assumptions that, given the key parameters of the system, lead to the optimum design in terms of the minimum number of RF readers. Section 4 relates to the measurement system for the speed detection of a battery-less BLE tag. It demonstrates how RF WPT and a battery-less BLE tag can implement a measurement system for speed detection with time domain readouts and the ability to share the information (speed) through IoT mechanisms. Section 5 describes the system setup, the experimental results, and their correlation to the data obtained in the design phase. Section 6 reports the conclusions.

## 2. System Description

**Figure 1:**
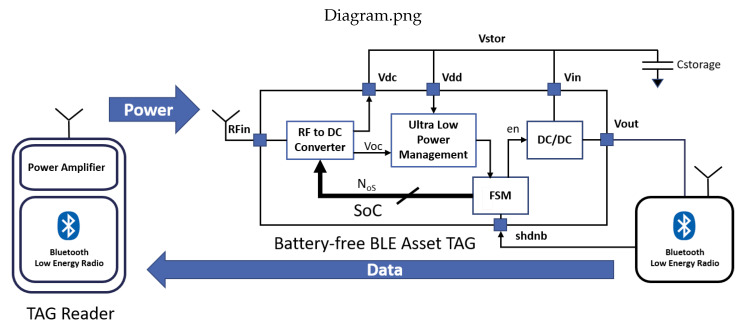
RF wireless power transfer system.

Long distance RF wireless power transfer (WPT) is used to remotely power a battery-less BLE asset tag. Figure 1 shows the block diagram of the proposed system. The architecture is based on a dual frequency system with two distinct frequencies used for WPT and data communication. For remote power transfer, the reader and the tag use a carrier centered on the unlicensed 868 MHz industrial, scientific, and medical (ISM) band. For data communication, the reader and the asset tag operate over an 80 MHz bandwidth centered inside the 2.4 GHz ISM band. The choice of the reader frequency for the power transfer is very important and is dictated by a compromise between size constraints for both the tag and the reader and minimizing free space path loss (FSPL). These two requirements indeed oppose each other, as the dimensions of the tag are largely due to the antenna, which is inversely proportional to the working frequency, which in turn directly impacts the FSPL. As the equation for Friis transmission [68] shows, for typical power transmission in the 868 MHz band in free space, the transmitted power decays −30 dB (1/1000) after a distance of only one meter and continues decaying −20 dB every 10 m. In comparison, choosing the 2.4 GHz frequency for the reader would result in a decay of −40 dB (1/10,000), or an order of magnitude more, over a distance of only one meter. This highlights that RF WPT is an inherently inefficient method of energy transfer and continued research into new architectures and design parameter selection is required. Notwithstanding these premises, RF energy transfer still represents a convenient way to supply energy to small energy devices such as IoT and wireless sensor nodes [54,69,70]. A BLE radio was used for data communication, as the system requires an ultra-low-power radio which is compatible with the specifications relating to the quantity of data to be exchanged and the communication data rate. In addition, the operating frequency of the BLE radio allows the design of very small antennas. The implementation of the reader consists of a very low-power RF sub-GHz transceiver (Spirit1 provided by STMicroelectronics) equipped with a power amplifier to deliver up to 27 dBm of output power and a very low power BLE radio that acts as signal receiver (the Bluetooth Low Energy SoC, compliant with Bluetooth 5.0 specifications, BLUENRG-2 provided by STMicroelectronics). The architecture of the tag system consists of two integrated circuits (ICs). A system on chip (SoC) designed specifically for this application receives and converts RF power into electrical power. The same BLE radio used for the reader is used for data communication. The SoC that functions as the power receiver is the key to system performance, and it will be shown mathematically that the PCE and sensitivity performance characteristics of the RF to DC converter are crucial in determining the number of readers. Higher performance obviously leads to a lower number of readers, which lowers the cost of the overall system. The SoC used in this application is a 2 μW self-powered IC. It integrates a wide-bandwidth (350 MHz to 2.4 GHz) RF to DC power converter with a maximum PCE of 37% at 868 MHz, an input power of −18 dBm and maximum output voltage of 2.4 V. The performance in terms of quiescent current of the ultra-low-power management is fundamental for the system sensitivity. Figure 1 reveals the architecture of the SoC. It consists of an RF to DC converter, ultra-low-power management, a digital finite state machine (FSM), and a DC/DC converter. The RF power is captured by an external antenna which is tied to the RFin input pin of the SoC. The RF to DC converter transduces the RF power into DC power and charges the external $C_{storage}$ capacitor through the output pin $V_{dc}$. Further, the RF to DC converter produces the voltage $V_{oc}$; this is a DC open circuit voltage used to indirectly measure the RF input power. The $V_{oc}$ and $V_{dc}$ voltages are the input of the ultra-low-power management, which powers and drives the FSM. The three units, RF to DC converter, the ultra-low-power management, and the FSM work in a closed loop. Depending on the received RF power level, indirectly measured through the Voc signal, the digital signal bus $N_{os}$, can be updated in real time to select the correct number of operating stages (CMOS voltage duplicators) of the RF to DC converter. The loop formed by the RF to DC converter, the ultra-low-power management, and the FSM, performs the conditions to get the maximum power point tracking (MPPT) while the RF input power level varies. This concept will be discussed in more detail in Section 3. From a functional point of view, the SoC converts the RF power received by the reader into a DC voltage Vdc that charges the storage capacity $C_{storage}$; the lower the quiescent current, the greater the net current transferred to the storage capacitor for the same amount of received power. The SoC integrates a nano-power circuit with a quiescent current as low as 75 nA, which allows scavenging power as low as 2 μW.

**Figure 2:**
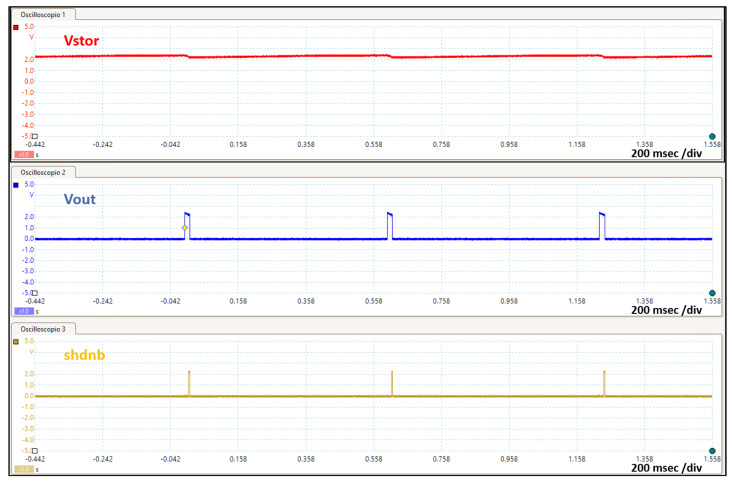
System on chip functional signals.

**Figure 3:**
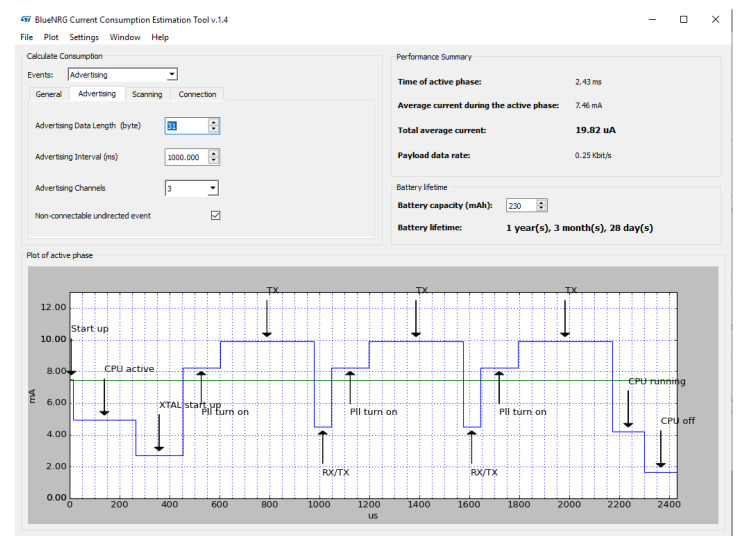
Bluetooth Low Energy (BLE) current consumption.

Figure 2 shows three different complete BLE advertising transmissions where the data packet is sent over three different BLE advertising channels. The BLE device is configured to transmit a 32 byte advertising data packet with a transmitted power of −14 dBm in the non-connectable, undirected advertising mode. In this operating mode the BLE device is not connected to any network and it is configured to broadcast information of any kind, ranging from, ambient data (temperature, air pressure, humidity, and so forth) to micro-location data (asset tracking, retail, and so forth) or orientation data (acceleration, rotation, speed, and so forth) [71]. While the tag is receiving energy from the reader, the storage capacitor is charged and its voltage $V_{stor}$ increases until it reaches the maximum value $V_{h}$. At this point, the ultra-low-power management enables the DC/DC converter to power the BLE device through the voltage $V_{out}$. While the voltage $V_{out}$ is above the minimum value (1.8 V) that keeps the BLE device on, this is active and broadcasts information. Therefore, the $C_{storage}$ capacitor inevitably discharges since the required current is much higher than the current generated from the RF signal. In fact, as shown in Figure 3, the $C_{storage}$ capacitor is required to supply a peak current to the BLE device in the order of milli-amperes, which is much higher than the harvested current that is typically in the order of micro-amperes.

**Figure 4:**
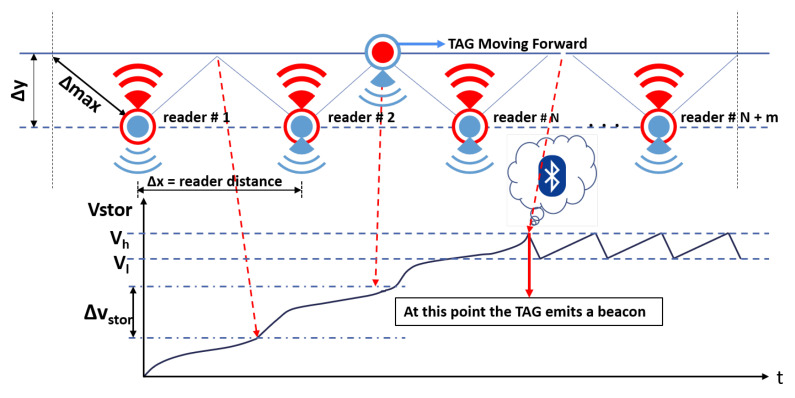
Wireless power transfer and $V_{stor}$ evolution.

As soon the BLE device ceases activity, it pulls up the “shdnb” signal, which triggers the internal finite state machine (FSM) of the SoC to reset the “en” signal, causing the DC/DC converter to turn off and the voltage $V_{out}$ to drop. Since the voltage $V_{out}$ is down and the BLE device is no longer biased, the “shdnb” signal also goes down. This limits the drop of energy stored in the $C_{storage}$ capacitor to the energy requirements of the BLE device, which may vary according to the length of the advertising data packet and the output transmitted power for which the BLE device is configured. For instance, if a BLE device is biased at the average voltage of 2 V, configured to operate in the non-connectable, undirected advertising mode to transmit an advertising data packet of 32 bytes with a power of −14 dBm, the estimated active phase time is 2.4 msec, the estimated average current during the active phase is 7.5 mA, and the estimated energy for the transmission is ≈ 36 μJ. If the transmitted output power is increased to +8 dBm, the estimated active phase time does not change, as it depends only on the advertising data packet length, while the estimated average current during the active phase increases to 13.4 mA and consequently the energy for the transmission rises to ≈ 65 μJ. The length of the advertising data has also an impact on the required energy of the BLE transmission. If the BLE device is configured to transmit an advertising data of 16 byte, with a transmitted power of −14 dBm, the estimated active phase time is reduced to 2 msec and the average current during the active phase is 7 mA with an estimated energy consumption of ≈ 28 μJ. Since the voltage drop of $V_{stor}$ is always kept to a minimum regardless of how the BLE was configured, the system can switch to scavenging energy earlier and thereby minimize the duty-cycle. This is a unique feature provided by the SoC, which can establish closed loop communication with any IoT node [72]. In this particular case study, the environment is a typical dynamic asset tracking system, where the assets travel at a certain speed *v* relative to the readers. It is important to keep in mind that in this context the tag is not static, and the received power cannot be considered constant. Therefore, depending on the energy required by the BLE radio to emit an advertising data packet, the average power $P_{av}$ that charges the $C_{storage}$ capacitor and the speed *v* of the moving tag, the node must cross a certain number of readers to accomplish the initial start-up, when the voltage $V_{stor}$ rises from 0 V to its maximum voltage $V_{h}$. It is worth noting that, since the tag is moving and the power $P_{av}$ is not constant, during the initial start-up, the voltage $V_{stor}$ will not rise continuously, but with a certain number of voltage steps. Figure 4 shows the behavior of the voltage $V_{stor}$ during the initial start-up and in steady-state. The figure shows a tag which is moving forward but it is worth highlighting that the direction of the tag is not relevant in the wireless power transfer process. Depending on the energy required by the BLE radio to emit a beacon, the available RF power at the receiver, and the speed of the moving tag, it can be observed that the node must cross a certain number of readers to accomplish the initial start-up. After that, the $C_{storage}$ capacitor is alternately charged with current provided by the RF power signal and discharged by the BLE radio current—both currents being very unbalanced. The following section deals with the design of the system, including some design insights, and discusses how to derive the size and minimum number of readers for a given system specification in terms of energy required by the BLE radio and the tag speed. It also discusses the impact of RF to DC performance in terms of sensitivity and PCE.

## 3. System Design

The main purpose of the investigation described in this article is to minimize the cost of the infrastructure, which is directly related to the number of readers to be installed. Figure 4 shows that the number of readers $N_{oR}$ necessary to complete the initial start-up, depends on the maximum value $V_{h}$ that the voltage $V_{stor}$ can reach and on the increase $\Delta V_{stor}$ it undergoes every time it passes through a reader distance Δx, as expressed by the following equation:

(1) $$N_{oR}=\frac{V_{h}}{\Delta V_{stor}}$$

The increment $\Delta V_{stor}$ of the voltage $V_{stor}$ depends on the average current $I_{avg}$ supplied by the RF to DC converter and the time Δt it takes for the tag to traverse the reader distance Δx, as expressed by the following equation:

(2) $$\Delta V_{stor}=\frac{I_{avg}\;\cdot \;\Delta t}{C_{storage}}$$

where $C_{storage}$ is the storage capacitor. In asset transport systems, the speed *v* of the objects is kept constant; therefore, it can be assumed that:

(3) $$\Delta t=\frac{\Delta x}{v}$$

According to Equation (3), Equation (2) can be expressed as:

(4) $$\Delta V_{stor}=\frac{I_{avg}\;\cdot \;\Delta x}{C_{storage}\;\cdot \;v}$$

Finally, Equation (1) can be expressed as:

(5) $$N_{oR}=\frac{V_{h}\;\cdot \;C_{storage}\;\cdot \;v}{I_{avg}\;\cdot \;\Delta x}$$

**Figure 5:**
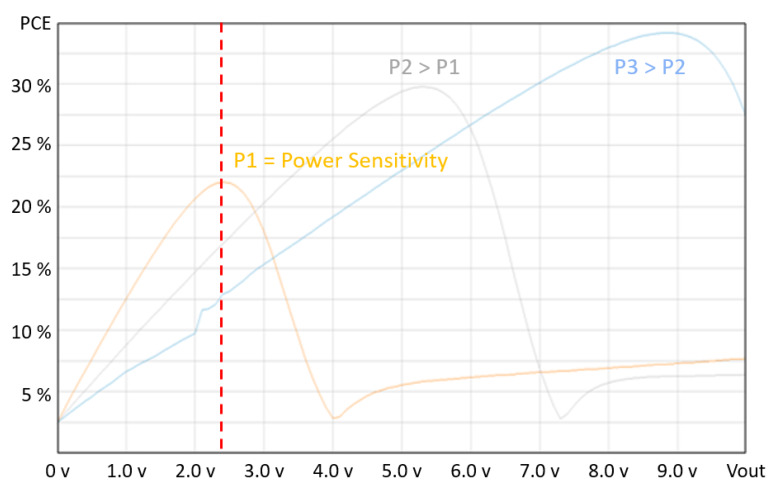
Power conversion efficiency (PCE) vs. DC output voltage of a static RF to DC converter.

Indeed, Equation (5) provides a useful relationship between the underlying parameters affecting system performance. It provides useful insights for the design of the key parameters of the system, which can help the designer choose the best system architecture for optimal performance. The equation suggests that for given values of the storage capacitor $C_{storage}$, the maximum value $V_{h}$ of the voltage $V_{stor}$, and the tag speed *v*, the optimum performance is achieved by maximizing the product $I_{avg}*\Delta x$. Both parameters $I_{avg}$ and Δx are related to the design and architecture of the RF to DC converter. In fact, $I_{avg}$ is the average current provided by the RF to DC converter and its value is related to the PCE performance, so the higher the PCE, the higher the values of the current $I_{avg}$ for a given transmitted power. Δx depends on the sensitivity performance of the RF to DC converter, so the higher the sensitivity performance, the higher the distance between the readers. In order to minimize the number of readers, both the sensitivity and PCE performance must therefore be maximized. Wireless Power Transfer in asset tracking systems requires dealing with very different power conditions. In fact, depending on the distance between the reader and the tag, the antenna orientation, and the transmission channel, the power spectrum can range from extremely low to relatively high, and more critically, may be subject to random variations in the available input power. In the system studied in this paper, the asset tag manages high variation in received power while moving across the readers: it is subject to very low power when it is at the far-end of the reader range to increasingly higher power levels as it gradually approaches. For these systems, the standard RF to DC converter architectures that only optimize sensitivity performance when the asset tag is relatively distant from the reader are not suitable. For the same reason, solutions optimizing the PCE performance only at a certain input power level, while providing good results when the tag is close to the reader, are also insufficient. These solutions can certainly provide optimal results in static working conditions, where the distance between the reader and the tag is fixed and known, but not in dynamic working conditions. Unfortunately, with typical RF to DC circuit architectures, it is difficult to optimize both parameters at the same time because the sensitivity and PCE characteristics tend to work against each other. Thus, dynamic systems require the ability to dynamically track the available power over a wide range using MPPT techniques [73,74,75,76,77,78]. These techniques share the common requirement of having to measure input power, which is not a straightforward task in an ultra-low-power environment, as this functionality inevitably consumes further power and contributes to further reducing the PCE of the system. This is also the reason why it is often difficult to determine whether an MPPT circuit is worthwhile when the power to harvest is very low. On this topic, an innovative technique has been proposed in [79], which shows how the received input power can be efficiently and dynamically tracked by monitoring the output DC open circuit voltage of a replicated and unloaded generic harvester such as an RF to DC converter. In its typical form, a CMOS RF to DC converter consists of a series of cascaded voltage doublers; i.e., a classic 2-stage Dickson charge pump [80]. In order to meet the required sensitivity power level, several stages need to be used. Moreover, the PCE performance of the circuit is generally maximized at a given input power level $P_{in}$ that is very close to, or in most cases, the same as the sensitivity power level. The output DC voltage is kept fixed by the system and the maximum permitted voltage is normally used. If the output voltage is kept fixed, however, and the number of stages $N_{oS}$ remains unchanged, as the input power increases, the circuit no longer works in optimal conditions and loses efficiency, as shown in Figure 5, which refers to a system with a 6-stage RF to DC converter powered with three different RF power levels: P1 = −18 dBm (sensitivity power level), P2 = −12 dBm, and P3 = −6 dBm.

**Figure 6:**
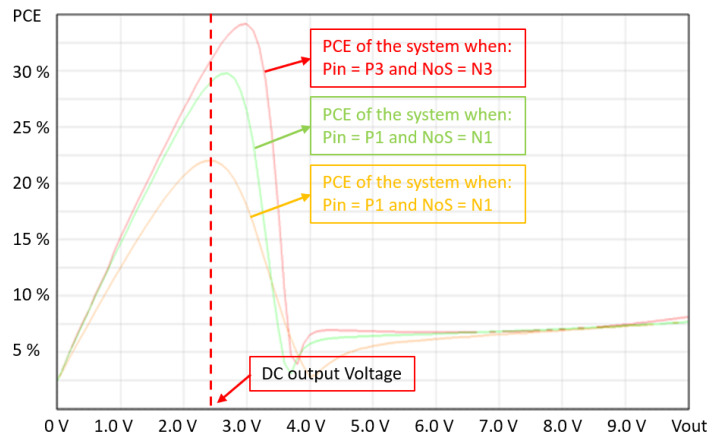
PCE vs. DC output voltage of a dynamic RF to DC converter.

Therefore, as shown in Figure 6, in order to maintain maximum sensitivity performance and at the same time restore and optimize the PCE, it is necessary to change the number of stages $N_{oS}$ of the converter based on our knowledge of the input power $P_{in}$. Moreover, Figure 6 shows three different setups of the RF to DC converter with three different numbers of stages, N1 = 6, N2 = 4, and N3 = 2. When the number of stages is the highest $N_{oS}$ = N1 = 6, the PCE is maximum at the lowest input power $P_{in}$ = P1 = −18 dBm. If the power is increased to $P_{in}$ = P2 = −12 dBm, the maximum PCE is achieved by reducing the number of stages to $N_{oS}$ = N2 = 4. When the input power is further increased to $P_{in}$ = P3 = −6 dBm, to obtain the highest PCE, the number of stages must be reduced to $N_{oS}$ = N3 = 2.

**Figure 7:**
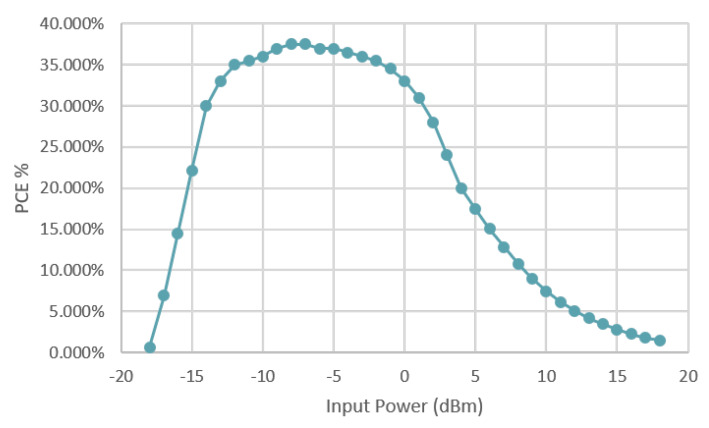
PCE vs. input power at 868 MHz.

The RF to DC converter used in the described system was designed to operate at 868 MHz according to the described guidelines. The optimal number of stages of the RF to DC converter is defined through the digital signal $N_{oS}$ issued by the finite state machine (FSM) circuitry, as shown in Figure 1. The ultra-low-power management measures the input received power through the open circuit voltage $V_{oc}$ signal. Thanks to these features, the best trade-off between sensitivity and PCE performance can be achieved. Figure 7 shows the results of the characterization of the RF to DC in terms of PCE vs. the input received power.

The instantaneous current that charges the $C_{storage}$ capacitor when a battery-free BLE tag crosses a reader distance Δx is not constant, but rather a function *Idc*(*x*) of the mutual distance x between the reader and the tag. The average charging current $I_{avg}$ received by the battery-free BLE tag while crossing a reader distance Δx is therefore given by the equation:

(6) $$I_{avg}=\frac{1}{\Delta x}\int_{0}^{\Delta x}Idc\left(x\right)dx$$

**Figure 8:**
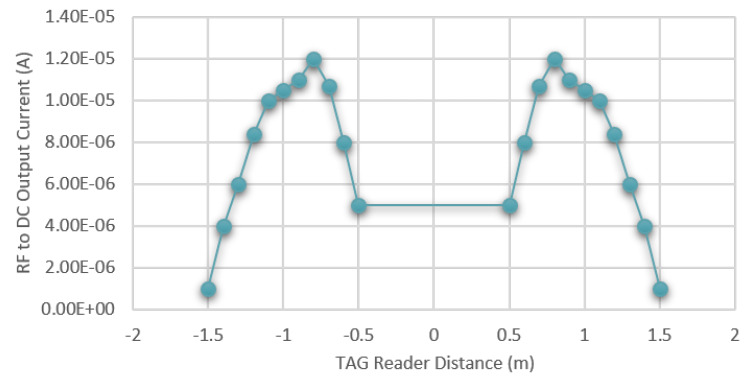
RF to DC output current vs. distance between tag and reader @ 868 MHz.

*Idc*(*x*) is the instantaneous current received, which depends on the transmitted power, the gain of both receiving and transmitting antennas, the minimum and maximum distance Δy and Δmax between the reader and the node, the operating frequency, and the PCE of the RF-to-DC converter. Figure 8 shows the characterization of the RF to DC converter in terms of the received instantaneous current *Idc*(*x*) versus the distance x in a typical application case where the readers are placed at the minimum distance Δy of 0.5 m and the sensitivity of the RF to DC converter allows a maximum distance Δmax of 1.5 m. The characterization has been performed at 868 MHz with the reader programmed to emit 27 dBm of power. Both the power transmitter and the RF energy harvester are equipped with the Revie Pro antenna provided by Laird [81].

## 4. Speed Detection

This section deals with the speed detection of an asset equipped with a battery-less BLE tag while moving at constant speed *v* through an asset tracking system. The scenario is the same as that illustrated in Figure 4, where the asset tag moves through several aligned and evenly distributed RF readers. The speed *v* of the tag can be determined thanks to the following equation:

(7) $$v=\frac{N_{oR}\;\cdot \;I_{avg}\;\cdot \;\Delta x}{V_{h}\;\cdot \;C_{storage}}$$

Equation (7) shows how the speed of the asset can be estimated from the number of readers crossed $N_{oR}$, at the time the BLE tag issued the first data packet, since all the other parameters, such as $V_{h}$, $I_{avg}$, Δx, and $C_{storage}$ are determined during the design phase of the system.

In a practical implementation of the system, this is equivalent to knowing the order number of the reader that has received the data transmitted by the battery-less BLE tag after it has accomplished the initial start-up. This can be achieved by acquiring the number of the reader whose BLE radio has received the highest received signal strength indication (RSSI). The RSSI can also be used with the transmitted power level information included in the BLE advertising data packet to determine the path-loss of the signal and identify how far the device is by using the following equation:

(8) Pathloss=Tx_power−RSSI

This result can help to optimize the costs of a system transporting assets at a constant speed, such as a conveyor belt. The advantage is that a special sensor is not necessary to detect the speed of the object, since this information is an inherent system parameter. In fact, the speed at which the asset is transported can be estimated through the knowledge of the RSSI detected by the reader and the number of readers traversed when the tag transmits for the first time. Therefore, this system can simultaneously provide identification, speed detection, and control through the simple implementation of RF WPT between BLE-equipped readers and a battery-less BLE asset tag, without the need for an actual speed sensor.

## 5. Experimental Results

The described system has been experimentally implemented and tested. The practical implementation has specified the minimum distance Δy = 0.4 m. The characterization results of the SoC show an average current of 1 μA at a maximum distance Δmax = 1.5 m, which according to Equation (9), provides a distance Δx between readers of 2.9 m.

(9) $$\Delta x=2\;\cdot \;\sqrt{{\left(\Delta max\right)}^{2}-{\left(\Delta y\right)}^{2}}$$

The BLE IC of the tag is biased at the voltage of 2 V and configured to transmit in the non-connectable, undirected advertising mode, with an advertising data packet of 32 bytes and an output power of −14 dBm. As already discussed, that results in an estimated energy consumption $E_{BLE}$ of the BLE of ≈ 36 μJ to be stored in the $C_{storage}$ capacitor. As Equation (10) revels, in order to minimize the value of the $C_{storage}$ capacitor, the maximum value $V_{h}$ of the voltage $V_{stor}$ is chosen as high as possible, and the minimum value $V_{l}$ is chosen as low as possible. For this reason, the value $V_{h}$ = 2.4 V is defined by the maximum operating voltage allowed by the 130 μm CMOS technology adopted to the design the SoC. The value $V_{l}$ = 2 V is defined to bias the BLE IC with a regulated voltage of 1.8 V and to allow a voltage headroom of 200 mV to the power stage of the DC/DC converter.

(10) $$C_{storage}=\frac{2\;\cdot \;E_{BLE}}{V_{h}^{2}-V_{l}^{2}}=\frac{2\;\cdot \;36\;\mu J}{2.4^{2}-2^{2}}=40\;\mu F$$

**Figure 9:**
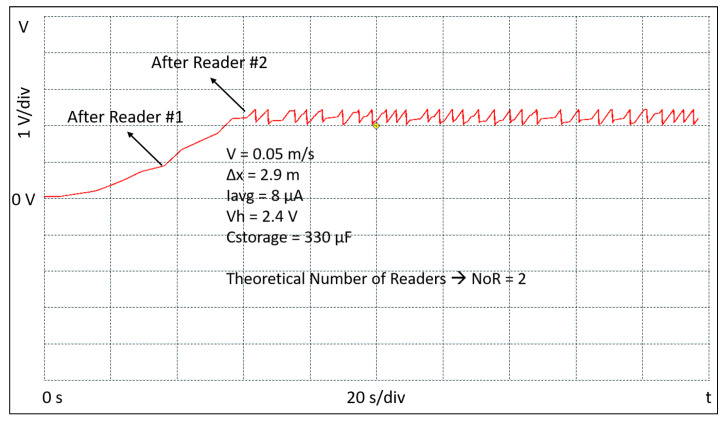
Experimental result of the tag crossing the readers at the speed of 0.05 m/s.

**Figure 10:**
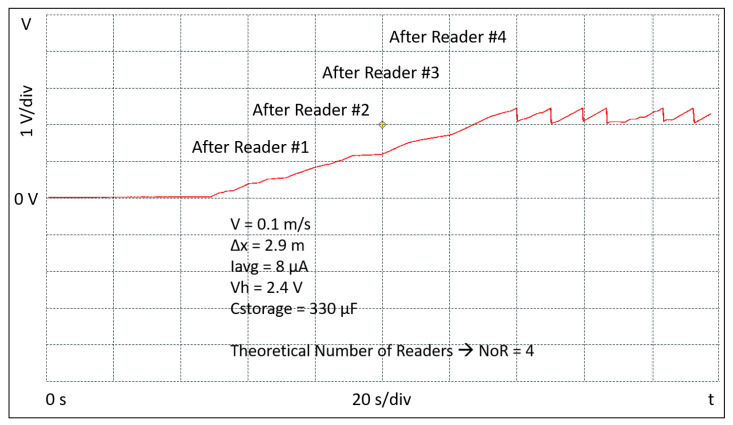
Experimental result of the tag crossing the readers at the speed of 0.1 m/s.

**Figure 11:**
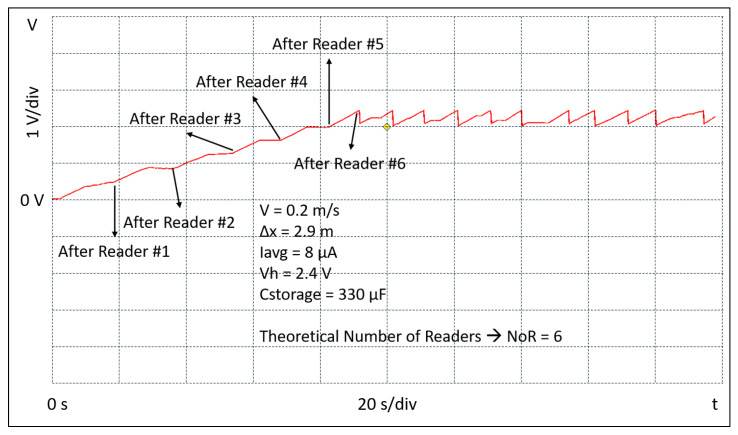
Experimental result of the tag crossing the readers at the speed of 0.2 m/s.

In order to allow some margin and extra energy capability to optionally activate other embedded sensors, a 330 μF $C_{storage}$ capacitor is used in the setup of the tag. The experimental setup consists of four readers, a portable oscilloscope, a robot, and the battery-free BLE tag. The readers are placed in a square and positioned so that the neighbors are equidistant at 2.9 m. Each reader is programmed to transmit a power of 27 dBm. During the measurements the tag is connected to a portable oscilloscope; it is continuously kept at the distance Δy and moved, by means of a robot, parallel across the readers and at constant speed. Several sessions of measurements have been performed at the three different constant speeds of 0.05 m/s, 0.1 m/s, and 0.2 m/s, respectively. The waveforms shown in Figure 9, Figure 10 and Figure 11 report the evolution of the voltage $V_{stor}$ during the initial start-up and beyond. They have been extracted from one of the measurement sessions and show the experimental data acquired through the oscilloscope. The figures also show the conditions in which the experiments have been conducted in terms of the tag speed *v*, the distance between the readers Δx, the value of average current supplied by the RF to DC converter $I_{avg}$, the value of the maximum $V_{stor}$ voltage $V_{h}$, and value of the $C_{storage}$ capacitor. Further, in the figures, the number of readers $N_{oR}$ theoretically obtained by applying Equation (5) is reported. The results show a good correlation with what has been experimentally measured. It can also be observed that during the initial start-up, the voltage $V_{stor}$ will not rise continuously, but with a certain number of voltage steps depending on the speed at which the tag is moved. Since the tag is continuously moving through the four readers, after the initial start-up, the tag keeps charging and transmitting. The charge/discharge pattern appears to be irregular and not periodic because the tag is moving through the readers and the instantaneous current that charges the $C_{storage}$ capacitor varies. Therefore, it can be noted that when the tag is nearby the reader, the slope of the voltage $V_{stor}$ is quite sharp, while when the tag is away from the reader the slope is slower. This discontinuity in the charging current causes the effect of an irregular and not periodic charge/discharge pattern, on the contrary to what it normally happens when a tag is charged through WPT while static. The figures confirm what was anticipated by Equation (5). The higher the speed *v*, the higher the number of the readers $N_{oR}$ needed to accomplish the initial start-up phase which, in an asset tracking system, is the event at which a tracked asset is identified for the first time. Its ID is finally transmitted by the TAG and received by the reader, and eventually deployed through a WSN.

**Figure 12:**
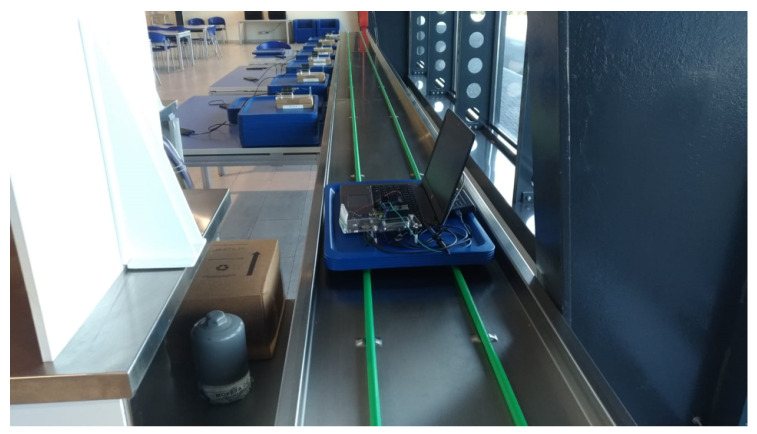
Experimental setup: placement of readers and the tag on the conveyor belt probed by the oscilloscope.

**Figure 13:**
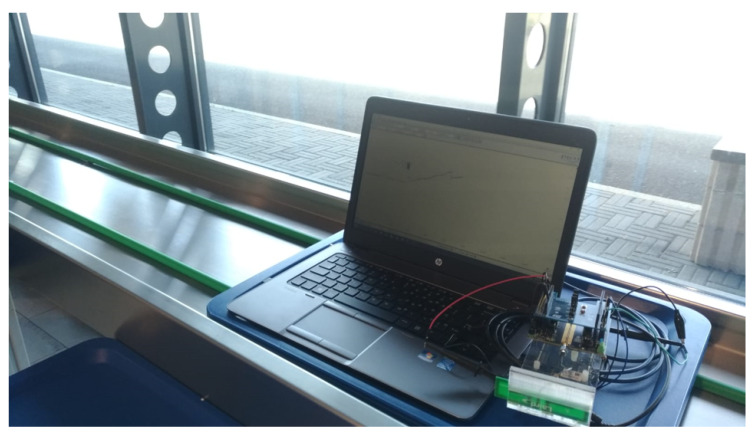
Experimental setup: tag on the conveyor belt probed by the oscilloscope.

The system has also been validated in a real environment involving an industrial conveyor belt. The experimental setup consisted of a conveyor belt, six portable readers, a battery-free BLE tag, and a portable oscilloscope. The conveyor belt was 18 m long; the six readers were programmed to continuously transmit the power of 27 dBm and placed along the conveyor belt, equally spaced at Δx = 2.9 m and Δy = 0.4 m, as shown in Figure 12. The tag shown in Figure 13 was probed by a portable oscilloscope. It was moved back and forth to cross the readers until the initial start-up phase was accomplished. In the first experiment the tag mounted a $C_{storage}$ capacitor of 330 μF and it accomplished its initial start-up phase after crossing 33 readers in good agreement with Equation (5). In the second experiment the $C_{storage}$ capacitor was reduced to 100 μF, and to accomplish the initial start-up phase it needed to cross 13 readers still in fair agreement with what predicted by Equation (5). The experiments have been repeated three times, confirming the same results.

## 6. Conclusions

In this paper, a system based on RF WPT to power a battery-less BLE tag for asset tracking was thoroughly presented with the aim of finding useful design insights and optimal solutions to minimize the number of RF readers. In order to achieve this, a system architecture based on WPT and BLE communication was chosen. A mathematical model providing a relationship to derive the minimum number of readers $N_{oR}$ from the most relevant system parameters such as maximum voltage $V_{h}$, sensitivity, and PCE of the RF to DC converter, as well as the speed and energy requirements of the tag, was provided. A design method was developed and applied to estimate the minimum number of readers. The mathematical model also provided design insights and guidelines for the specific circuit architecture of the RF to DC converter that was purposely designed and characterized. In addition, the mathematical relationship between the speed of a tracked battery-less BLE asset tag and the number of readers was provided. Finally, a real system was tested to demonstrate the agreement between experimental results and the proposed models, and to confirm the validity of the suggested approach for both calculating the minimum number of readers and detecting the speed.
